# Sexually transmitted Human Papillomavirus type variations resulting in high grade cervical dysplasia in North-East North Dakota and North-West Minnesota

**DOI:** 10.1186/1743-422X-3-46

**Published:** 2006-06-15

**Authors:** Lata Balakrishnan, Ryan Clauson, Timothy Weiland, Michelle Bianco, Barry Milavetz

**Affiliations:** 1 Department of Biochemistry and Molecular Biology, University of North Dakota, Grand Forks, ND, USA; 2 School of Medicine, University of North Dakota, Grand Forks, ND, USA; 3 Department of Pathology, University of North Dakota, Grand Forks, ND, USA; 4 School of Medicine, University of Iowa, IA, USA

## Abstract

**Background:**

A review of Pap smear diagnoses from a reference laboratory in Grand Forks, North Dakota over a 3-year period (07/00 to 10/03) revealed a two-fold higher rate of high grade squamous intraepithelial lesion in a community in northwest Minnesota (Roseau, 0.486%) than in northeast North Dakota (Grand Forks, 0.249%), in spite of both having similar rates of low-grade squamous intraepithelial lesion (1.33% vs.1.30% respectively)

**Objectives:**

To identify the different types of HPV present in patient populations showing high-grade dysplasia in Grand Forks, ND and Roseau, MN.

**Study design:**

Formaldehyde-fixed paraffin-embedded cervical tissue samples were analyzed using polymerase chain reaction (PCR) to detect the presence of HPV type 16, 18 and 31.

**Results:**

Our studies showed that 41 % of samples from Roseau were triply infected with HPV serotypes 16, 18 and 31 in comparison to 12 % from Grand Forks.

**Conclusion:**

Due to the small sample size we were unable to prove the study to be statistically significant. However, our results suggest that the presence of HPV 16, 18 and 31 in triply infected samples may be the cause of the higher percentage of high-grade dysplasia in Roseau, MN when compared to Grand Forks, ND.

## Background

Human Papillomavirus (HPV), a member of the papovavirus family, is a small circular double stranded DNA virus with a genome of approximately 8 Kb. HPV causes the most common sexually transmitted disease (STD) in the U.S. with at least 5.5 million new infections each year and an actively infected population of approximately 20 million people [1]. There are more than 100 different genotypes of HPV, which are known to cause a wide range of infections including common warts, genital warts, recurrent respiratory papillomatosis, cervical dysplasia and cervical cancer. Fifteen HPV types are classified as high-risk types {16, 18, 31, 33, 35, 39, 45, 51, 52, 56, 58, 59, 68, 73, and 82} and twelve are classified as low-risk types {6, 11, 40, 42, 43, 44, 54, 61, 70, 72, 81, and CP6108} [2]. HPV has been found in 99.7 % of cervical carcinomas worldwide with HPV 16 and 18 the predominant genotype in these carcinomas. [3]. The virus has been postulated to gain entry into the body through microscopic abrasions of the surface epithelium most often followed by integration of the viral genomes of the high-risk types into basal cells late in infection and subsequent transformation of the basal cells.

During an analysis of the severity of cervical dysplasia in patients attending clinics in Grand Forks, ND and Roseau, MN, we observed that the number of patients with high-grade dysplasia was approximately twice as high in Roseau compared to Grand Forks [0.249% and 0.486% respectively; (p < 0.004)] in spite of similar rates of low-grade dysplasia [1.304% and 1.332% respectively] in both these areas. Grand Forks, ND and Roseau, MN are geographically related areas separated by approximately 100 miles. Since none of the typical risk factors including age of 18–28, pregnancy, smoking, high school diploma or less, use of oral contraceptive pills, or presence of coexisting STD (including condylomata acuminata) correlated with the increased incidence of high grade dysplasia, we hypothesized that the increased incidence might be a result of differences in the high-risk HPV types responsible for the infections. The aim of this study was to use polymerase chain reaction (PCR) to identify HPV types 16, 18 and 31 present in patient populations showing high-grade dysplasia in Grand Forks, ND and Roseau, MN.

## Materials and methods

### Study population

Archival paraffin-embedded, formalin-fixed cervical tissue samples from patients diagnosed with high-grade dysplasia were obtained from Altru Clinic, Roseau, MN and Altru Clinic, Grand Forks, ND over a three year period from 07/00 – 10/03. Grand Forks represented the control group, while Roseau, MN represented the experimental group. Statistical significance was analyzed by Chi square test and confirmed by z test using Sigma Stat software.

### HPV type analyses

DNA from formaldehyde-fixed paraffin-embedded tissues was extracted using the thermal cycler deparaffinization method as previously described [4] with minor modifications. Extracted DNA preparations were first subjected to PCR targeting a 155 base pair fragment (GP 5+/GP6+) of the L1 open reading frame (ORF) of HPV [5]. The HPV types in the positive samples were characterized by PCRs specific for HPV types 16, 18, and 31 {Primer Sets used, Type Specific 16 [6], Type Specific 18 [7], Type Specific 31 [8]. The final 30 μl of PCR mixture contained 2.5 μl sample, 2.0 mM MgCl_2_, 3 μl of 10X PCR Gold Buffer, 200 μM deoxynucleoside triphosphates, 50 pmol of each primer (IDT Oligos) and 0.5 μl AmpliTaq Gold Polymerase (all reagents were purchased from Applied Biosystems, Foster City, CA). The amplification conditions were set to 1 min of denaturation at 95°C, 2 min of annealing at 40°C and 1.5 min of extension at 72°C for 40 cycles. HeLa and CaSki cells were used as positive controls for HPV 18 and HPV 16 respectively. The presence of an appropriately sized amplification product was monitored by gel electrophoresis and ethidium bromide staining.

## Results and discussion

**Figure 1:**
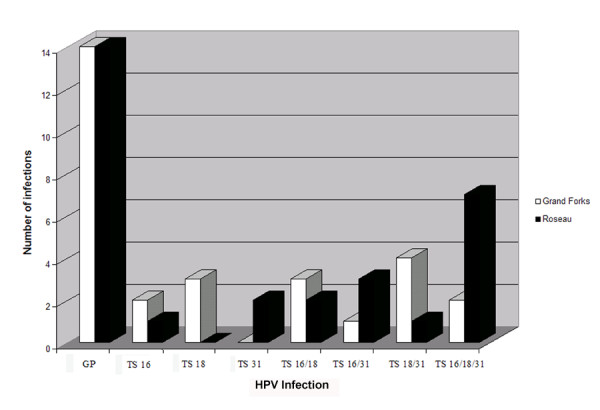
**HPV Infection in Grand Forks, ND (control group) versus Roseau, MN (experimental group)**. Cervical tissue samples from patients from Grand Forks, ND (control group) and Roseau, MN (experimental group) were analyzed for the presence of different high-risk HPV infections. All samples were first analyzed with general primers (GP) and subsequently analyzed with type-specific (TS) primers. Varying incidence of type-specific HPV infections are presented as a comparison between infection in Grand Forks, ND and Roseau, MN.

Out of the total thirty-four high-grade cervical dysplasia tissues analyzed by PCR with the general primers (GP 5+/GP 6+) targeting a 155 base pair fragment of the L1 open reading frame of HPV, twenty-eight tested positive. Samples from four normal patients, which were used as negative controls, did not show any evidence of HPV infection. Since the general primers have previously been reported to be less sensitive than specific primers in screening for certain high-risk HPV types [9], all of the samples were then amplified with primer sets specific to each of the three different high-risk types (HPV 16, 18 and 31) which are known to be associated with cervical carcinoma (Walboomers et al., 1999). Of the 17 cases studied from Grand Forks – control group, 14 samples (82 %) were positive for the general primers (Figure 1). Two samples (12 %) and 3 samples (18 %) were positive for only HPV types 16 and 18 respectively. There were no cases with single infection with HPV types 31. Three samples (18 %) showed dual infections with both HPV 16 and 18, 1 sample (6 %) were doubly infected with HPV 16 and 31 and 4 samples (24 %) were doubly infected with HPV 18 and 31. Two samples (12 %) showed triple infections with HPV 16,18 and 31. One sample tested negative with general primers but tested positive with HPV 18 specific primers.

Of the 17 cases studied from Roseau – experimental group, 14 samples (82%) were positive for the general primers (Figure 1). Single infections of HPV 18 were not detected. One sample (6%) had single infections with HPV 16 and 2 samples (12 %) with HPV 31. Two samples (12%) were doubly infected with HPV 16 and 18, 3 samples (18 %) with HPV 16 and 31 and 1 sample 6 % with HPV 18 and 31. Triple infections with HPV 16,18 and 31 were detected in 7 samples (41 %) cases. One sample tested negative with general primers but tested positive with HPV 31 specific primers.

We also analyzed four squamous cell carcinoma tissue samples from Roseau, MN and all the four samples tested positive with the general primers for HPV. We further analyzed these samples for the presence of specific types of HPV and found that three out of the four samples were triply infected with HPV type 16, 18 and 31. One sample contained a double infection with HPV 16 and 31.

Differences in the incidence of cervical high-grade dysplasia in two separate communities within the same geographic area were correlated with the presence of multiple HPV type infections and differences in the HPV type infecting the dysplastic cells. Multiple infections with different HPV types have been previously reported to be associated with high-grade dysplasia [10,11]. Infection with HPV 16 has also been known to cause high-grade squamous intraepithelial lesions which progress into malignancy [12]. However, in our study population we found that the presence of single or double infections with HPV 16 did not alone appear to contribute to the higher incidence rate of high-grade dysplasia. Our results suggest that the presence of HPV types 16 and 18 along with HPV 31 in the triply infected samples may be responsible for the higher rate of HSIL in the experimental population. Supporting this hypothesis was the observation that 3 of 4 samples of cervical squamous cells carcinoma from area patients also were triply infected with HPV 16, 18, and 31 suggesting that the presence of multiple infections along with HPV 16 might play a significant role in the progression of low-grade dysplasias to high-grade dysplasia. However due to the small sample size tested, this data precludes any statistical significance. Although we analyzed all of the samples obtained from Roseau MN over a three year period, we realize that this analysis was limited by the relatively small number of samples which could be obtained from this location. However, it was quite interesting that major differences in HPV type infecting cervical tissue could exist in distinct localities within the same geographic area. Similarly since we only analyzed for HPV 16, 18 and 31, we do not know if other viral types were present in single or multiple infections. Confirmation of the role of triple infections by HPV types in causing high-grade dysplasias will require further molecular studies in a larger risk population.

## Competing interests

The author(s) declare that they have no competing interests.

## Authors' contributions

LB conducted the HPV analyses and wrote the manuscript. RC helped in the HPV PCR analyses. TW provided the archival tissue samples. MB was instrumental in getting the project started and doing some background research. BM coordinated the research efforts at UND and edited the manuscript. All co-authors read and approved the final manuscript.
